# Rhizobacterial compositions and their relationships with soil properties and medicinal bioactive ingredients in *Cinnamomum migao*

**DOI:** 10.3389/fmicb.2023.1078886

**Published:** 2023-02-16

**Authors:** Lixia Li, Xuedong Yang, Bingli Tong, Deng Wang, Xiu Tian, Jiming Liu, Jingzhong Chen, Xuefeng Xiao, Shu Wang

**Affiliations:** ^1^Forest Ecology Research Center, College of Forestry, Guizhou University, Guiyang, Guihzou, China; ^2^Guizhou Extension Station of Grassland Technology, Guiyang, Guizhou, China; ^3^School of Life Sciences, Guizhou Normal University, Guiyang, Guizhou, China; ^4^College of Urban and Rural Construction, Shaoyang University, Shaoyang, China

**Keywords:** *Cinnamomum migao*, soil rhizobacteria, soil chemical properties, medicinal bioactive ingredients, redundancy analysis

## Abstract

**Introduction:**

Rhizobacterial communities and their metabolites can affect plant growth, development, and stress resistance, as well as the biosynthesis and accumulation of bioactive compounds in medicinal plants. This relationship has been well-characterized in many medicinal herbs, although much less commonly in medicinal trees.

**Methods:**

Here, we analyzed the composition and structure of *Cinnamomum migao* rhizobacterial communities across nine growing regions in Yunnan, Guizhou and Guangxi, China, as well as differences in soil properties and fruit bioactive compounds.

**Results:**

Results showed that the *C. migao* rhizobacterial communities exhibited high species richness, but location-specific differences in structure. Site-specific differences in soil properties and bioactive compounds were also observed. Furthermore, rhizobacterial community compositions were correlated with both soil properties and fruit bioactive compounds, metabolism-related functions were most common in *C. migao* rhizobacteria.

**Discussion:**

Several bacterial genera, including *Acidothermus*, *Acidibacter*, *Bryobacter*, *Candidatus_Solibacter*, and *Acidimicrobiales*, potentially promote the biosynthesis and accumulation of 1,8-cineole, cypressene, limonene, and α-terpineol, *Nitrospira* and *Alphaproteobacteria* may play an inhibitory role. Finally, our results emphasized the critical role that soil pH and nitrogen levels play in driving rhizobacterial community structure, and specific functional bacteria can also counteract with soil properties, *Acidibacter* and *Nitrospira* can affect soil pH and nitrogen effectiveness. Overall, this study provides additional insight into the complex correlation of rhizosphere microorganisms with bioactive ingredients and soil properties of medicinal plants.

## 1. Introduction

Rhizosphere microorganisms are often considered “the second plant genome” because these microbes are so crucial for plant nutrition, growth, development, yield, secondary metabolite biosynthesis, and defense (Berendsen et al., 2012; Chaparro et al., 2012; Philippot et al., 2013; Qu et al., 2020; Wigginton and Amador, 2020). Both the community structure and diversity of rhizomicroorganisms are impacted by soil environmental conditions, including soil physicochemical properties, plant root exudates, and accumulated humus (Delgado-Baquerizo et al., 2018). Of these, soil physicochemical properties appear to be the primary drivers of rhizomicrobial community structure, likely because these properties pose the greatest constraints on microbial growth (Dunbar et al., 2002; de Carvalho et al., 2016). Because of this, soil microbial communities are highly variable among geographic locations and microclimates.

Bacteria are the most abundant rhizosphere denizens, particularly in nutrient-rich soils, and are vitally important for maintaining microecological stability (Marschner et al., 2001; Yang et al., 2019). Not only do rhizosphere bacteria and their metabolites affect the growth, development, and stress resistance of plants, but they can also impact plant metabolism and synthesis of bioactive compounds (Huang et al., 2018; Zhang et al., 2020). Rhizosphere bacteria can also regulate plant gene expression through the inducer effect. Specifically, microbes release small molecules into the rhizosphere and when plants recognize these chemical signals, it results in changes to both metabolism and gene expression, and the production and accumulation of plant secondary metabolites (Vives-Peris et al., 2020). This process is particularly important, from a human health perspective, in medicinal plants, where microbial induction leads to the bioaccumulation of medicinal bioactive compounds and other compounds of interest (Onrubia et al., 2013; Xie et al., 2019). However, the study of the relationship between medicinal plant metabolism and rhizosphere microorganisms is in its infancy.

While the majority of medicinal plant research is focused on roots and rhizomes, seeds are the second most important sources of medicinal and therapeutic compounds (Chinese Pharmacopoeia Commission, 2020). Additionally, the majority of medicinal plant research is focused on herbs and lianas of Asteraceae, Lamiaceae, Araliaceae, Apiaceae, Ranunculaceae, and Campanulaceae (Manero et al., 2012; Arora et al., 2016; Zeng et al., 2018; Xie et al., 2019; Li et al., 2021; Zhang et al., 2022), with some research on trees in Taxaceae (*Taxus*), Nyssaceae (*Camptotheca acuminata*), and Meliaceae (*Dysoxylum binectariferum*; Onrubia et al., 2013; Solaiman and Anawar, 2014). Although the subject of much less research, up to 20% of medicinal and therapeutic compounds are derived from trees (Chinese Pharmacopoeia Commission, 2020). There is a paucity of research regarding the correlation between rhizomicrobial community dynamics and changes in the bioactive constituents of medicinal plants, although some generalizations might be made. For example, medicinal plants with connected roots or rhizomes may experience more direct and intense microbial induction effects, while aerial plant parts, such as leaves, fruits, or flowers, may be less affected by rhizosphere activities. Additionally, herbs and some lianas may have much shorter lifespans than shrubs or trees, and the time of colonization of the recruited bacteria is inconsistent，potentially altering their relationship with and response to rhizomicrobes. Therefore, we may speculate that the magnitude of importance of either the direct or indirect effects of rhizomicrobes on the accumulation of bioactive compounds in medicinal plants is likely to be tissue-, contact-, time-, and environment-dependent, and highly variable across species, landscapes, and geographies.

*Cinnamomum migao* H. W. Li, endemic to southwestern China, bears fruit with high medicinal value. Specifically, *C. migao* fruit has been used in the treatment of coronary heart disease, angina pectoris, and asthma, among other conditions (Chen et al., 2021). However, due to high demand, the supply of *C. migao* fruit is experiencing a shortage. Therefore, understanding the ecological adaptability of this species and finding reliable methods to improve the yield and quality of *C. migao* fruit is of considerable importance. In this study, (a) we sought to characterize the relationships between soil chemical properties, rhizomicrobial community structure, and *C. migao* fruit bioactive compounds across several growing regions. (b) By understanding how soil chemistry interact with microorganisms, (c) and how microbial properties impact the accumulation of bioactive compounds in medicinal plants, growers can implement methods to increase the yield and quality of medicinal plant materials.

## 2. Materials and methods

### 2.1. Materials

*Cinnamomum migao* H. W. Li belongs to the family Lauraceae (*Cinnamomum*), which is an aiphyllium. It is a small population species, concentrated in the narrow area at the junction of Guizhou, Guangxi and Yunnan in China, its interpopulation gene differentiation coefficient (Gst) is 0.2636, the gene flow at the population level (Nm = 1.3968) > 1, and the gene flow between the populations of *C. migao* is confirmed to be large and the genetic differentiation is small (Li et al., 2018). The materials of *C. migao* in this experiment are wild-type.

### 2.2. Collection of soil and fruit samples

**Figure 1:**
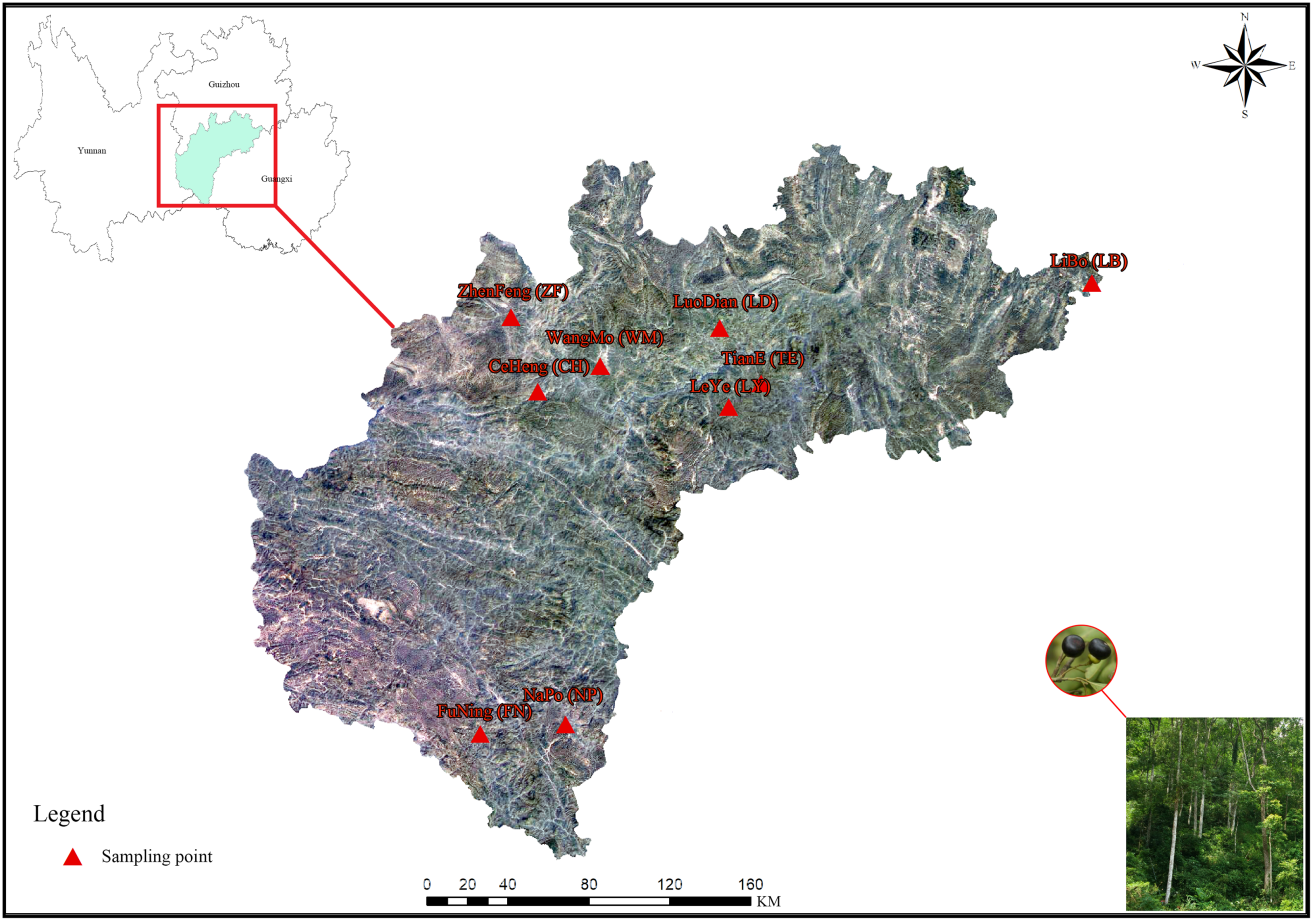
Map of the *Cinnamomum migao* sample collection sites. The map comes from the standard map service system of the Ministry of natural resources, PRC (http://bzdt.ch.mnr.gov.cn/), which is authorized for free. The picture in the right corner was taken by the first author during field investigation. The nine *C. migao* sampling points at the junction of Guizhou Province, Guangxi Province, and Yunnan Province, China.

Rhizosphere soil and fruit samples were collected in October, 2018, from 5 healthy, wild *C. migao* trees (diameter 32–38 cm) at each of 9 sampling sites in Guizhou Province, Guangxi Province, and Yunnan Province, China. During the sampling process, the basic information such as sampling location, longitude, latitude and altitude were indicated. Information of the sampling sites are shown in Figure 1 and Table 1.

**Table 1:** Geographic and meteorologic information of each collection site.

Sample site	Latitude	Longitude	Altitude(m)	Annual average temperature (°C)	Average relative humidity (%)
LuoDian County, Guizhou Province (LD)	25°17′18”	106°35′43”	790	19.7391	72.0095
WangMo County, Guizhou Province (WM)	25°7′13”	106°3′53”	670	20.2872	69.1774
CeHeng County, Guizhou Province (CH)	25°0′21”	105°47′11”	750	19.2473	70.6263
ZhenFeng County, Guizhou Province (ZF)	25°20′15”	105°40′6”	990	17.9782	71.5254
LiBo County, Guizhou Province (LB)	25°29′19”	108°15′2”	660	18.8502	73.0736
TianE County, Guangxi Province (TE)	25°2′33”	106°46′44”	1,120	19.6655	72.3385
LeYe County, Guangxi Province (LY)	24°56′17”	106°38′6”	940	19.6846	72.0917
FuNing County, Yunnan Province (FN)	23°29′6”	105°31′53”	1,190	19.7667	71.8118
NaPo County, Yunnan Province (NP)	23°31′40”	105°54′39”	680	19.8442	71.7932

The geographic and meteorologic information for each site, including Annual Average Temperature (AAT) and Average Relative Humidity (ARH), obtained using inverse distance weighting (IDW) interpolation in ArcGIS 10.2.

To collect soil samples, after removing the humus from the soil surface, samples of healthy plant roots were collected at a depth of 0–20 cm along the base of each tree by using a drill. Soil samples were packed into the sterilized plastic bags respectively, and sampling records (time, place, number, etc.) were made on. Then they were quickly stored in ice boxes and brought back to the laboratory. Samples were processed as described by Beckers, the remaining soil was first manually removed by shaking the roots until approximately 0–2 mm soil was left attached to the roots, and then rhizosphere soils (100 g) were collected by shaking on a platform (20 min, 120 rpm; Beckers et al., 2017). The rhizosphere soil samples from 5 trees per site were mixed in equal proportions to form a single conglomerate sample (500 g; Shen et al., 2013). Homogenized soil samples were divided into two sub-samples using a 2-mm sieve: one subsample was dried at 45°C in a blast drying oven to a constant weight for determination of soil chemical properties; the other sample was stored in a − 80°C ultra-low temperature freezer for extraction of microbial DNA.

The *C. migao* fruits were harvested when it turns from green to brown or black (October). The collection of fruit and soil samples shall be consistent, and the corresponding fruits are taken separately and brought back to the laboratory. Fruits were dried at 45°C in a blast drying oven to a constant weight. The dried fruits was crushed into powder with multifunctional crusher, screened with 40-mesh sieve, and storaged at 4°C for determination of bioactive compounds (Sun et al., 2022).

### 2.3. Determination of soil chemical properties and fruit bioactive compounds

Soil chemical properties were determined according to the methods of Bao (2000). The potentiometric method was used to measure pH, water-soil ratio of 2.5:1(w/v); Organic matter (OM), Potassium dichromate oxidation volumetric method; Total nitrogen (TN), Alkaline hydrolysis diffusion method + semi micro Kjeldahl method; Total phosphorus (TP), Heating digestion method + Vanadium molybdate blue colorimetric method; Total potassium (TK), Heating digestion method + flame photometric method; Alkali hydrolyzed nitrogen (AN), Alkaline hydrolysis diffusion method + semi micro Kjeldahl method; Available phosphorus (AP), Sodium bicarbonate injection + Vanadium molybdate blue colorimetric method; Available potassium (AK), Ammonium acetate extraction + flame photometric method. The measured indicators of each soil sample (1–2 g) were measured five times in parallel, and the results were averaged.

Gas chromatography (GC) was used to quantify the bioactive compounds in *C. migao* fruit, including 1,8-Cineole, sabinene, limonene, and α-terpineol (Chen et al., 2021). The powder of *C. migao* samples was precisely weighed (1.00 g). Chromatographic conditions: the column was HP-5 capillary column (30 m × 320 mm, 0.25 μm); Carrier gas: high-purity nitrogen N_2_; Flow rate: 1 ml⋅min^−1^; Injection volume: 1 μl; Split ratio: 10:1, injector temperature: 200°C; temperature-rising program: the initial oven temperature was 50°C, and maintained for 3 min, raised the temperature to 85°C at 5°C⋅min^−1^ and held for 2 min, raised the temperature to 90°C at 5°C⋅min^−1^ and held for 2 min, raised the temperature to 160°C at 5°C⋅min^−1^ and held for 0 min, raised the temperature to 220°C at 20°C⋅min^−1^ and held for 0 min. Detector (FID) temperature: 235°C (Dai et al., 2013).

Analysis of variance (ANOVA) and Tukey T-test were employed to detect statistically significant differences (*p* < 0.05, *p* < 0.01) in these properties and compounds between samples using SPSS 22.0, Microsoft Excel 2010 and Origin 9.0 system software were used to analyze and map the data of soil chemical properties and fruit bioactive ingredients.

### 2.4. Extraction and sequencing of bacterial genome

The bacterial genomic DNA was extracted from of rhizosphere soil samples using the FastDNA^®^ Spin Kit, the method was carried out according to the kit instructions. Added up to 0.5 g soil to Lysing Matrix E tube. Secured tubes in FastPrep Instrument and process for 30 s at speed 5.5. Centrifuged Lysing Matrix E tube at 14,000 ×g for 30 s. Transferred supernatant to a clean tube. Added 250 μl PPS and mixed by shaking the tube by hand 10 times. Centrifuged at 14,000 ×g for 5 min to pellet precipitate. Transferred supernatant to a clean 15 ml tube. Added 1 ml **Binding Matrix Suspension** to the supernatant. Placed on a rotator or inverted by hand for 2 min to allow binding of DNA to matrix. Placed tube in a rack for 3 min to allow settling of silica matrix. Discarded 500 μl of supernatant. Resuspend **Binding Matrix** in the remaining amount of supernatant. Transferred approximately 600 μl of the mixture to a **SPIN**^**TМ**^ **Filter** and Centrifuged at 14,000xg for 1 min. Emptied the catch tube and added the remaining supernatant to **SPIN**^**TМ**^ **Filter** and spun again. Added 500 uL **SEWS-M** to the SPIN^TМ^Filter and Centrifuged at 14,000 ×g for 1 min. Decanted flow-through and replaced **SPIN**^**TМ**^ **Filter** in catch tube. Centrifuged at 14,000 ×g for 2 min to “dry” the matrix of residual **SEWS-M** wash solution. Removed **SPIN**^**TМ**^ **Filter** and placed in fresh kit-supplied **catch tube**. Air dried the **SPIN**^**TМ**^ **Filter** for 5 min at room temperature. Added 50 μl **DES** and gently stirred matrix on filter membrane with a pipette tip or vortex/finger flipped to resuspend the silica for efficient elution of the DNA. Centrifuged at 14,000 ×g for 1 min to transfer eluted DNA to **catch tube**. DNA was application-ready.

DNA quality was assessed by 1% agarose gel electrophoresis (5 V/cm, 20 min). The V4-V5 region of the bacterial 16S rDNA was used for high-throughput sequencing. Prior to PCR amplification, the 338F forward primer was combined with 0.8 μl ACTCCTACGGGAGGCAGCAG (5 μmol/l) forward primer adapter, and the 806R reverse primer was combined with 0.8 μl GGACTACHVGGGTWTCTAAT (5 μmol/l) reverse primer adapter. PCR amplification was carried out in a 20-μL reaction system: 5× Buffer (4 μl), 2.5 mmol/l dNTPs (2 μl), TransStart Fastpfu DNA Polymerase (0.4 μl), template DNA (10 ng), plus ddH_2_O to 20 μl. The PCR reaction was carried out according to the following parameters: pre-denaturation at 95°C for 5 min, denaturation at 95°C for 3 min, annealing at 55°C for 3 s, 29 cycles; extension at 72°C for 10 min, hold at 10°C (ABI GeneAmp 9,700). The PCR products were sequenced reagents on an Illumina HiSeq platform. Data processing and biodiversity analysis were performed on the I-Sanger bioinformatics analysis cloud platform.^1^

### 2.5. Sequence data processing

After the paired-end (PE) sequence data were obtained by HiSeq sequencing, the PE reads were spliced into one coding sequence according to the overlap relationship between PE reads. Low-quality reads and chimera sequences were filtered out, and effective sequences were distinguished according to the barcode and primer sequences (Caporaso et al., 2010). Clean reads were used to perform taxonomic and operational taxonomic unit (OTU) cluster analysis. The QIIME platform was used to cluster sequences with a similarity of ≥0.97 into OTUs, and the longest sequence of each OTU was selected as the representative sequence. The representative OUT sequences were aligned using the Silva database^2^ to obtain taxonomic information for each OTU. Species composition and redundancy analysis (RDA) were performed on the I-Sanger bioinformatics analysis cloud platform. The Mothur v.1.30.1 index analysis package^3^ was used to calculate alpha diversity indices, including Shannon-Wiener, Simpson, ACE, and Chao1.

### 2.6. Bacterial ecological functional analysis

PICRUSt software was used for functional prediction of 16S amplicon sequencing results. First, PICRUSt was used to normalize OTU abundance. Then, the Greengene ID of each OTU was used to obtain COG (clusters of orthologs groups) family and KEGG (Kyoto encyclopedia of genes and genomes) Ortholog (KO) information. The eggNOG database was used to obtain the functional abundance spectrum based on the COG database. The KEGG database was used to obtain pathway, KO, and EC information, as well as the abundance of each functional category. Finally, PICRUSt was used to obtain three levels of metabolic pathway information and pathway abundance.

## 3. Results

### 3.1. *Cinnamomum Migao* rhizobacterial community composition across regions

#### 3.1.1. Rhizobacterial alpha diversity

A total of 319,173 high-quality rhizobacterial sequences were obtained from samples spanning nine *C. migao* growing regions. From these, 2,112 distinct OTUs were obtained, with an average sequence length of 434 bp (Table 2). The coverage exceeded 99.00% across all nine growing regions, indicating that the results were valid and realistic. Standardized by the minimum number of sequences (27,740), both the Shannon and Simpson indices indicated that the WM site had the highest bacterial diversity while the LB site had the lowest. Specifically, the Shannon index indicated WM > CH > LY > FN > TE > LD > NP > ZF > LB and the Simpson index indicated LB > ZF > LD > TE > FN > NP > LY > CH > WM. According to the Chao1 and ACE indices, the WM and CM sites had the highest bacterial diversity, while the NP site had the lowest.

**Table 2:** Sequence statistics and rhizobacterial community alpha diversity indices obtained from samples spanning nine *Cinnamomum migao* growing regions.

Sample site	Valid sequences	OTUs	Mean length (bp)	Shannon index	Simpson index	ACE index	Chao1 index	Coverage
LD	35,789	1,199	432	5.7140	0.00894	1336.67	1370.07	0.9922
CH	42,882	1,430	434	6.0790	0.00608	1554.55	1550.45	0.9922
LB	30,500	1,052	439	5.3194	0.01788	1213.34	1221.51	0.9925
ZF	43,272	1,028	431	5.5604	0.00981	1163.11	1186.63	0.9932
NP	42,058	929	432	5.5898	0.00765	1034.34	1061.86	0.9944
TE	33,506	1,286	432	5.8709	0.00853	1443.25	1488.41	0.9915
WM	33,834	1,495	436	6.3143	0.00371	1639.59	1641.25	0.9915
FN	29,592	1,315	437	5.8773	0.00850	1458.47	1468.32	0.9920
LY	27,740	1,382	436	6.0523	0.00732	1533.30	1550.38	0.9919

Chao and Ace are used to evaluate the community richness, while Shannon and Simpson are used to assess the community diversity.

#### 3.1.2. Rhizobacterial community composition

**Figure 2:**
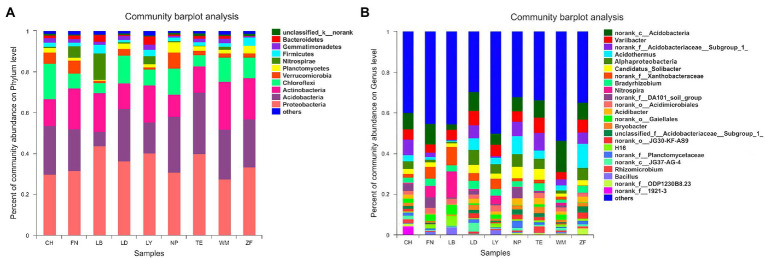
Rhizobacterial community structure across nine sample sites. Phylum-level **(A)** and Genus-level **(B)**. The color of the column represents the different genera, and the length of the column represents the proportion size of the genus. The species with <0.01% abundance were classified together as “others.” “norank” represent “unidentified” bacterial genus.

We detected 29 phyla, 63 classes, 129 orders, 251 families, and 387 genera of bacteria across all soil samples. As shown in Figure 2A, the rhizobacteria were most commonly represented by the phyla Proteobacteria (34.81%), Acidobacteria (21.85%), Actinobacteria (16.71%), Chloroflexi (10.16%), Verrucomicrobia (3.48%), Nitrospirae (2.87%), Planctomycetes (2.38%), Firmicutes (2.38%), Gemmatimonadetes (1.68%), and Bacteroidetes (1.64%; Average value). The relative abundance of the top four bacterial phyla (Proteobacteria, Acidobacteria, Actinobacteria, and Chloroflexi) was all greater than 10%, which was significantly higher than any other bacterial phyla. Additionally, site-specific differences in bacterial community composition were also observed. Proteobacteria was the dominant phylum across all nine sample sites, which order was LB (43.78%), LY (40.13%), TE (39.91%), LD (36.47%), ZF (33.35%), FN (31.65%), NP (30.69%), CH (29.69%) and WM (27.57%), followed by Acidobacteria at all sites except LB and LY, which order was TE (30.05%), NP (27.27%), LD (25.42%), WM (24.09%), CH (23.87%), ZF (23.34%) and FN (20.32%), and Nitrospirae (12.77%) in LB samples. As shown in Figure 2B, the rhizobacteria were most commonly represented by the genera *Acidobacteria* (unidentified; 8.39%), *Variibacter* (5.40%), *Acidobacteriaceae* subgroup 1 (unidentified; 4.68%), *Acidothermus* (4.59%), *Alphaproteobacteria* (3.99%), and *Candidatus-Solibacter* (3.18%; Average value). Again, site-specific differences in bacterial community composition were also observed. The dominant genus at both ZF (11.86%) and NP (8.70%) was *Acidothermus*, followed by *Acidobacteria* (unidentified), *Variibacter*, *Alphaproteobacteria*, and *Acidobacteriaceae* subgroup 1 (unidentified). The dominant genus at LB was *Nitrospira* (12.77%), followed by *Xanthobacteraceae* (unidentified; 9.02%), H16 (5.06%), and *Variibacter* (4.93%). The dominant genera at FN were *Acidobacteria* (unidentified; 10.25%), *Xanthobacteraceae* (unidentified; 5.72%), *Nitrospira* (5.57%), and DA101 soil group (unidentified; 5.16%). The dominant genus at TE (9.34%), LD (8.08%), WM (8.63%), CH (15.39%), and LY (5.57%) was *Acidobacteria* (unidentified), followed by *Alphaproteobacteria*, and *Variibacter*.

**Figure 3:**
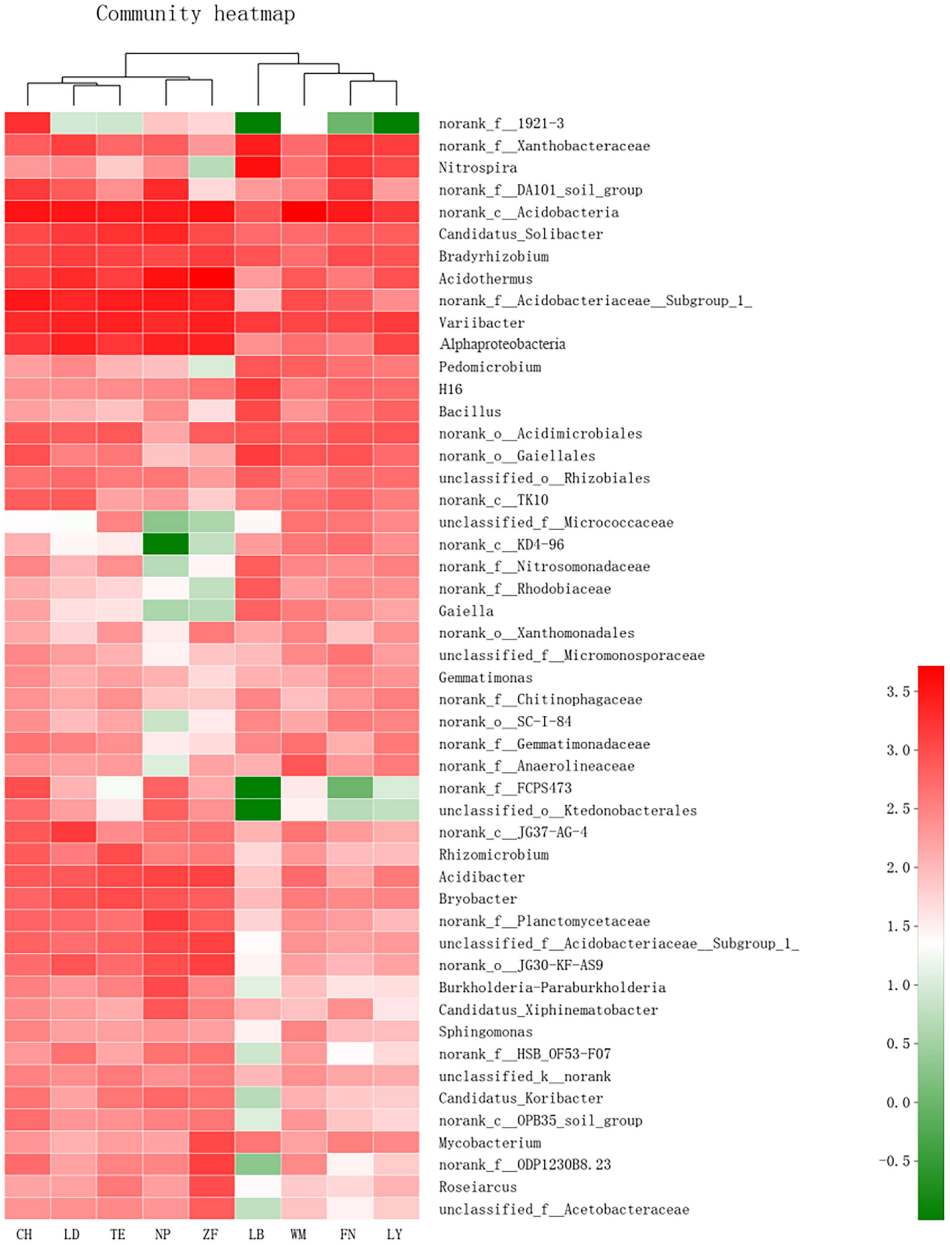
Heatmap of Bacteria in Rhizosphere Soil of *C. migao.* Heatmap of the top 50 most abundant genera in bacterial communities detected across nine sample sites. Dendrograms for hierarchical cluster analysis and sample locations were shown at the top.

As shown in Figure 3, Site-specific differences in the abundance of rhizobacteria community of *C. migao* were observed obviously at genus level. The rhizobacteria of the nine samples can be divided into two categories: LD and TE and CH first clustered together, and then clustered with NP and ZF as one class, and FN, LY and WM and LB as the other category; Among them, the rhizobacterial community of *C. migao* is most similar between LD and TE, and between ZF and NP.

#### 3.1.3. Rhizobacterial ecological functions

**Figure 4:**
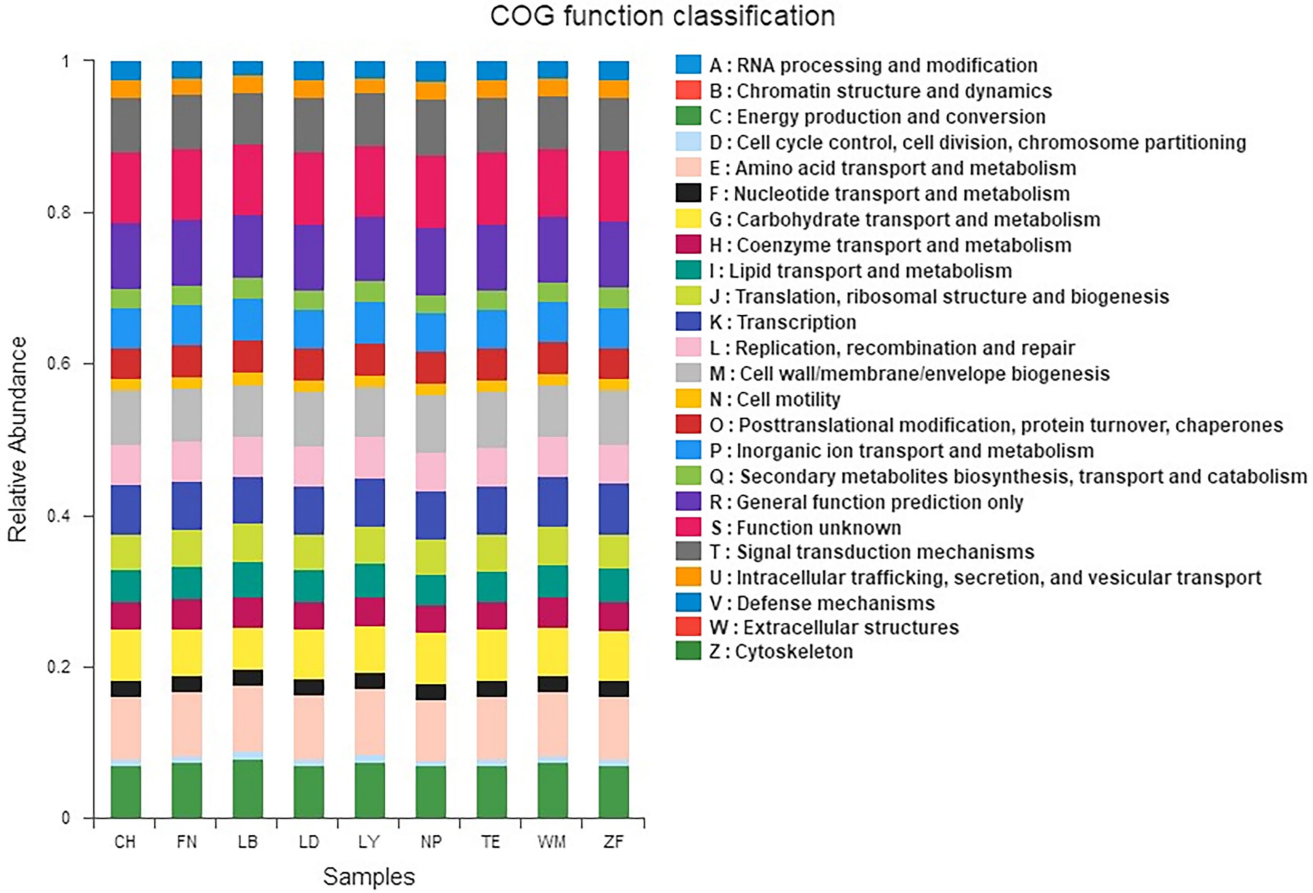
COG (clusters of orthologs groups) functional classification of the rhizobacterial communities across nine sample sites. The x-axis represents samples and the y-axis represents relative abundance presented as a percentage.

The rhizobacterial unigenes across nine samples sites were classified into 24 gene function families based on the COG database (Figure 4). The eight most common functional gene families were energy production and conversion, amino acid transport and metabolism, carbohydrate transport and metabolism, transcription, cell wall/membrane/envelope biogenesis, general function prediction, function unknown, and signal transduction mechanisms, together representing 57.64–63.49% of all gene functions. At the primary functional later, KEGG analysis found six categories of biological functions across all samples, including Cellular Processes, Environmental Information Processing, Genetic Information Processing, Human Diseases, Metabolism, and Organismal Systems. Among these, Metabolism, Environmental Information Processing, and Genetic Information Processing were most common, accounting for 51.32–52.71%, 12.32–14.55%, and 14.81–15.77% of all gene functions, respectively. At the secondary functional layer, 41 sub-functions were identified across all samples, including Amino Acid Metabolism, Carbohydrate Metabolism, Cell Motility, Cellular Processes and Signaling, Energy Metabolism, Lipid Metabolism, Membrane Transport, Poorly Characterized, and Replication and Repair, among others.

### 3.2. *Cinnamomum Migao* soil properties and bioactive compounds across regions

**Figure 5:**
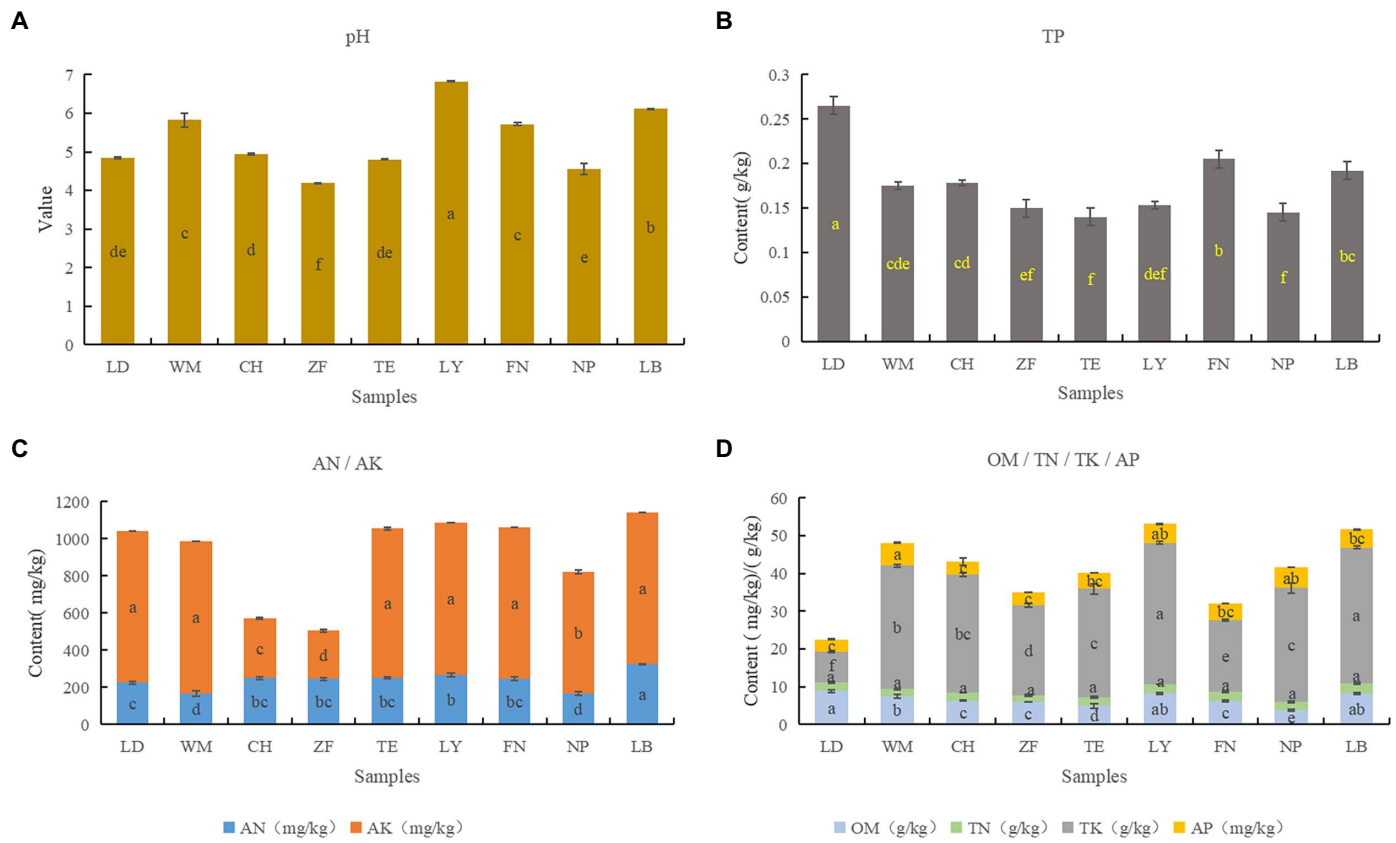
Nutrient and chemical properties of *C. migao* rhizosphere soils from nine sites. The ANOVA analysis and Turkey were used to compare the soil chemical properties across nine sample sites. Different letters indicate significant differences (*p < 0.05*; *n* = 5). The color of the column represents the different soil chemical properties, and the length of the column represents the content of the soil chemical property. **(A)** PH, **(B)** the content of total phosphorus (TP), **(C)** contents of alkali hydrolyzed nitrogen (AN) and total potassium (TK), **(D)** contents of organic matter (OM), total nitrogen (TN), available phosphorus (AP), and available potassium (AK).

The rhizosphere soil chemical properties were analyzed across all nine sample sites (Figure 5). Overall, the soil across all sites was found to be more or less acidic, with significant differences between sites. Across sites, significant differences were found in OM, AN, TP, TK, and AK, with small differences in AP, and no significant difference in TN. Correlation analysis between soil chemical factors showed that soil pH were positively correlated with the 7 soil nutrient indicators, especially with AK, AP and OM (*P < 0.01*); TK with TP and TP with OM were negatively related (*P < 0.01*; Table 3). Overall, both the pH and nutrient contents of LB and LY were higher than the other sites, with ZF having the lowest pH and nutrient content. Across all sites, the AP content tended to be low while the AK and AN contents tended to be high. According to the “Nutrient Classification Standard of the Second Soil Census of China,” across sites the AP content would be classified as grade 4 (5–10 mg/kg) or grade 5 (3–5 mg/kg), the AK content would be classified as grade 1 (>200 mg/kg), and the AN content would be classified as grade 1 (>150 mg/kg).

**Table 3:** Correlation of chemical Properties of Rhizosphere Soil of *C. migao.*

	PH	TK	TN	TP	AK	AN	AP	OM
PH	1							
TK	0.475^*^	1						
TN	0.402^*^	0.038	1					
TP	0.071	−0.699^**^	0.308	1				
AK	0.598^**^	−0.018	0.412^*^	0.285	1			
AN	0.371	0.180	0.435^*^	0.109	0.018	1		
AP	0.613^**^	0.361	0.084	−0.068	0.386^*^	0.020	1	
OM	0.541^**^	−0.129	0.152	0.601^**^	0.328	0.349	0.221	1

*. Significantly correlated at the 0.05 level (bilateral).**. Significantly correlated at the 0 0.01 level (bilateral).

**Figure 6:**
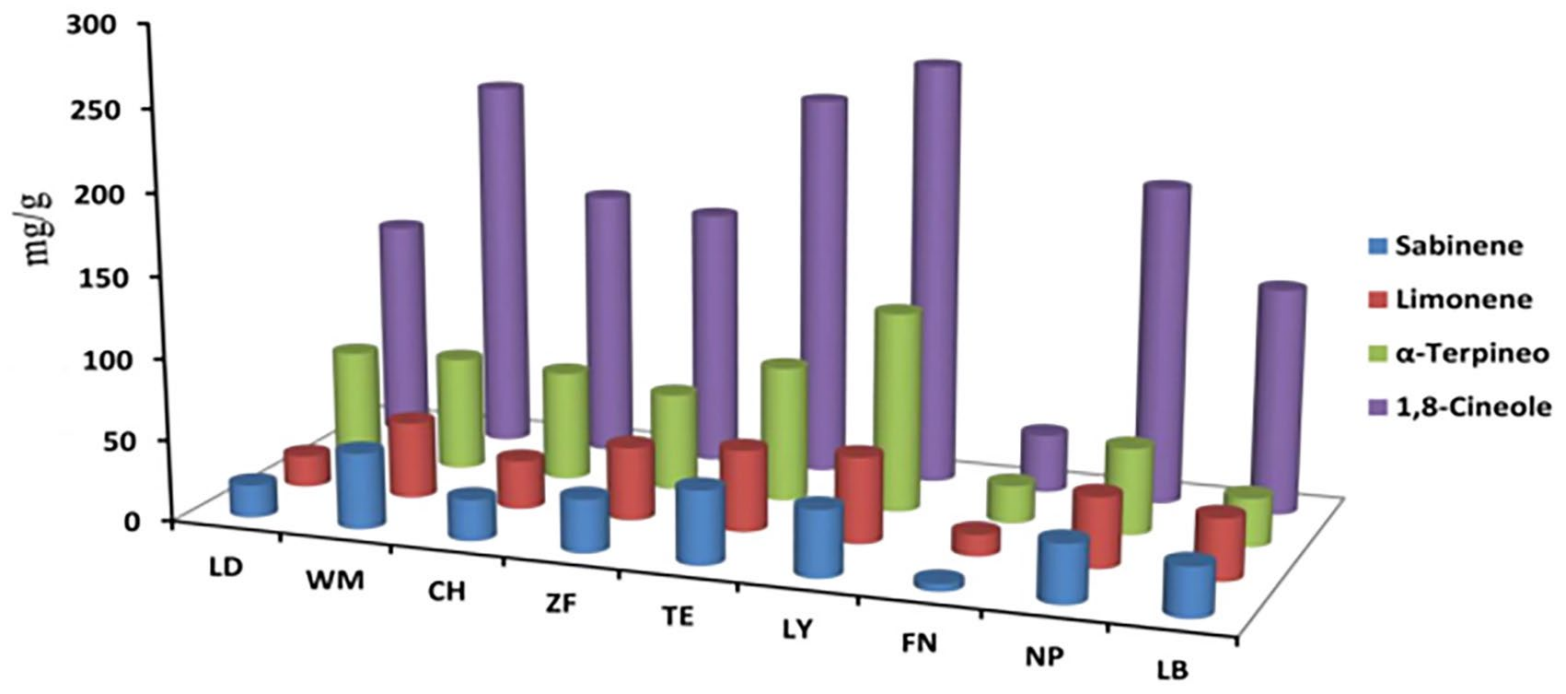
Content of medicinally important bioactive compounds of *C. migao* fruit across nine sites. The ANOVA analysis and Turkey were used to compare the soil chemical properties across nine sample sites. Different letters indicate significant differences (*p < 0.05*; *n* = 5). Four bioactive compounds, 1,8-cineole (blue), sabinene (orange), limonene (grey), and α-terpineol (yellow), the length of the column represents the content of the bioactive compound.

The content of several medicinally important bioactive compounds of *C. migao* fruit, including 1,8-cineole, sabinene, limonene, and α-terpineol, were determined by GC (Figure 6). The most plentiful compound was 1,8-cineole, followed by α-terpineol, sabinene, and limonene. Fruit from LY contained the highest content of 1,8-cineole, α-terpineol, and limonene, while fruit from FN contained the lowest content of all compounds. The differences in the content of bioactive compounds of *C. migao* fruit from different regions were considerable, suggesting that differences in environmental and soil characteristics translate into vast differences in bioactive compound accumulation.

### 3.3. Correlations between soil chemical properties, rhizomicrobial community structure, and *Cinnamomum migao* fruit bioactive compounds

**Figure 7:**
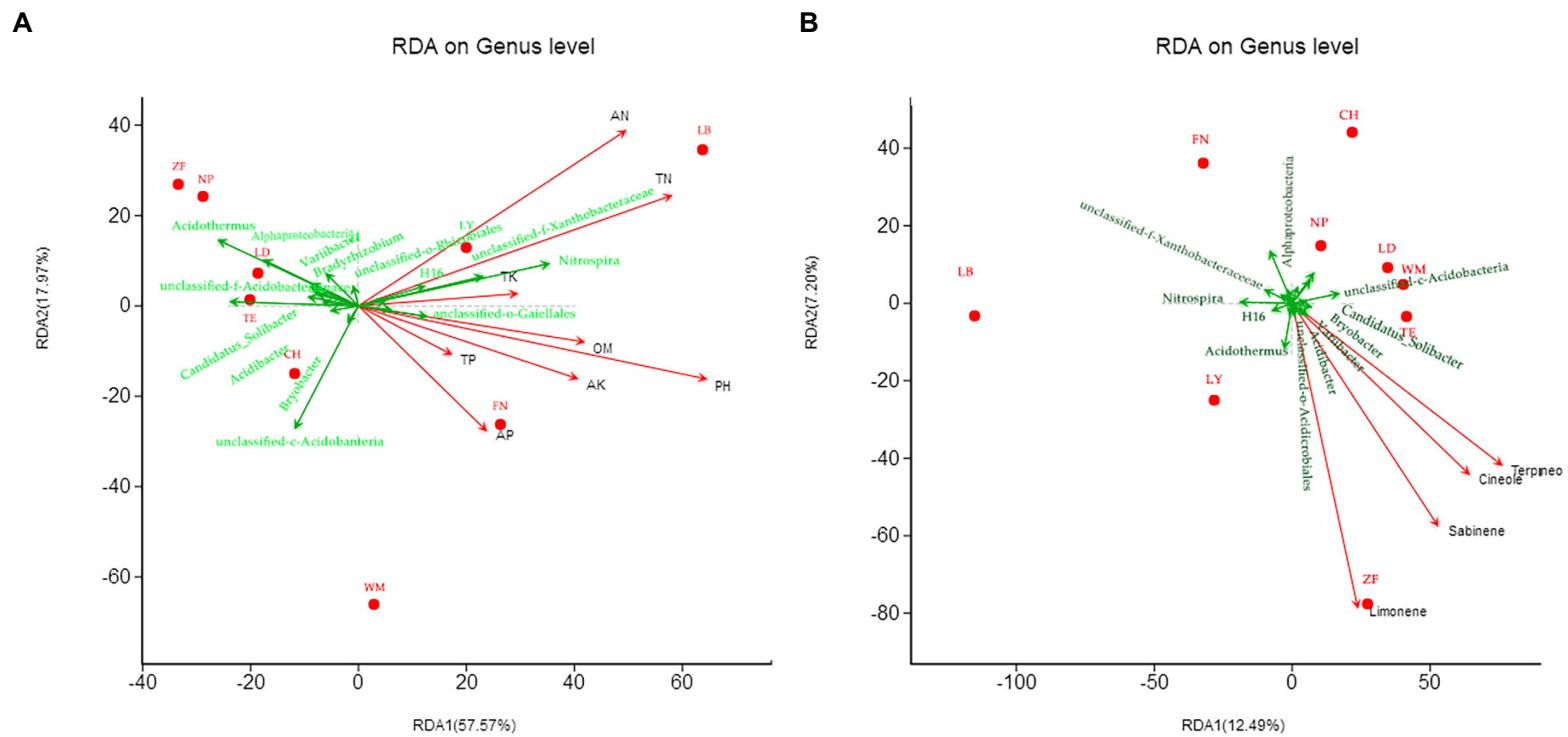
Canonical correspondence analysis (RDA) of rhizobacterial communities with soil chemical factors **(A)** and *C. migao* fruit bioactive compounds **(B)** across nine sites. The red dots represent different sample sites, the green arrows represent the top 20 abundance genera, and the red arrows represent soil chemistry and active ingredients, arrow length represents the degree of influence.

RDA was used to examine the relationships between the top 20 bacterial genera and soil chemical and nutritional properties (Figure 7A), with the horizontal axis as the first ranking axis (57.57% contribution) and the vertical axis as the second ranking axis (17.97% contribution). The bacterial genera *Nitrospira*, Xanthobacteraceae (unidentified), H16, and *Gaiellales* (unidentified) were positively correlated with all eight soil chemical indicators. *Rhizobiales* (unidentified) was positively correlated with AN, but not TN. *Bryobacter* and *Acidobacteria* (unidentified) were positively correlated with AP, but not TP. *Bradyrhizobium* was negatively correlated with seven soil chemical indicators. Most of the other bacterial genera, such as *Acidibacter*, *Acidobacteriaceae* subgroup 1 (unidentified), *Candidatus-Solibacter*, *Variibacter*, *Acidothermus*, *Acidimicrobiales*, and *Alphaproteobacteria*, were negatively correlated with the eight soil chemical indicators to different degrees. Additionally, pH, AN, and TN were strongly correlated, and together had the greatest influence on bacterial community composition at LB, LY, and FN.

RDA was also used to examine the relationships between the top 20 bacterial genera and the content of *C. migao* fruit bioactive compounds (Figure 7B), with the horizontal axis as the first ranking axis (12.49% contribution) and the vertical axis as the second ranking axis (7.20% contribution). The bacterial genera *Acidothermus*, *Acidibacter*, *Bryobacter*, *Candidatus_Solibacter*, and *Acidimicrobiales* (unidentified) were positively correlated with all four bioactive compounds, particularly, *Acidothermus* and limonene have a strong positive correlation. *Acidobacteria* (unidentified) was positively correlated with all bioactive compounds except limonene. *Nitrospira*, *Alphaproteobacteria* and *Xanthobacteraceae* (unidentified) were negatively correlated with all four bioactive compounds.

## 4. Discussion

### 4.1. Variability of soil properties, bacterial communities, and bioactive compounds

Analysis of the bacteria present in the rhizosphere of *C. migao* from different regions revealed rich and varied communities. The dominant genera of rhizosphere bacteria from different habitats were basically the same, although there are some differences in abundance. Overall, Proteobacteria (34.81%), Acidobacteria (21.85%), Actinobacteria (16.71%), and Chloroflexi (10.16%) were the dominant bacterial phyla across all field sites; the defined bacterial genera, including *Variibacter* (5.40%), *Acidothermus* (4.59%), *Alphaproteobacteria* (3.99%), *Candidatus_Solibacter* (3.18%), *Bradyrhizobium* (3.05%), *Nitrospira* (2.86%), *Acidibacter* (1.91%) and *Bryobacter* (1.88%) were found to be the dominant genera. In comparing with previous studies, there are some species with similar dominant bacterial genera to *C. migao*, such as: natural mixed forests, apple trees, *Abies Fabri*, *Robinia Pseudoacacia*, and *Cunninghamia Lanceolata* (Jiang et al., 2017; Yang, 2019; Zhang, 2019; Nie et al., 2020; Xiong et al., 2021). However, some species dominant-phylum are as similar as 90% to *C. migao*, the specific bacterial genera differ from our results, such as: *Taxus*, which is an arboreal medicinal plant (Hao et al., 2016). Additionally, some plants have different genotypes from *C. migao*, and their dominant taxa are distinct (Li et al., 2021). Studies have shown that plants with different genotypes may differ in their morphological characteristics, ecological habits, and physiological traits. There may also be differences in the characteristics of the rhizomicroorganisms they recruit. For example, (a) species with different ecological habits have more discrepancy’s in their colonizing environments, e.g., rice vs. maize, and their rhizomicrobes also differ significantly (Li et al., 2014; Nevita et al., 2018). (b) Plants with different phenotypes have disparate environmental effects, e.g., variances in the depth, number and size of plant roots makes distinct effects on soil amelioration, ultimately leads to differences in enriched rhizobacteria. (c) Plants with different physiological characteristics may differ in their metabolites, and the secretion and release of specific metabolites may inhibit some genera that are not adapted to the type and concentration of the products, while attract those who use the products as a nutritional feedstocks and structural materials. It is worth mentioning that legumes actively recruit rhizobia for forming a mutually beneficial symbiosis (Glyan Ko, 2015).

After comprehensive analysis, we found that plants with similar dominant genera to *C. migao* were mostly derived from forestland (*C. migao*’s samples also from forests), and these dominant genera reflected the bacterial structure characteristics in specific ecological environment, which differed from the rhizobacterial compositions of crops or herbs in farmland (Zhang et al., 2022). This may be a commonality, as arboreal (forestland) recruited bacteria which have longer colonization periods and more stable ecotope than herbs (farmland). Besides, because of the abundant diversities of bacteria and the more complex mechanisms of interaction between arbors and microorganisms, there are still a large number of undefined bacterial taxa in forests (Du et al., 2017; Zhang et al., 2022), more than half taxa are undefined in *C. migao* rhizobacteria, and the defined are basically rare. Researches on the composition and function of sylvatic microorganisms should be strengthened in the future.

Analysis of soil chemical and nutritional properties indicated that there were significant differences in pH, TK, TP, and OM between sites, while differences in AN, AK, and AP differed only slightly, and TN did not differ at all between sites. Both the pH and nutrient content of soils from LB and LY were higher, while ZF soils had the lowest pH and nutrient content. Furthermore, the contents of four medicinally important bioactive compounds (1,8-cineole, sabinene, limonene, and α-terpineol) from *C. migao* fruit were different significantly between sites, with fruit from LY, WM, and TE containing the highest content of bioactive compounds, the minimum was ZF. This may be related to G × E (genotype × environment) interactions, Studies have confirmed that different genotypes have genetic traits for variable chemical compositions. For example, the active constituents of *Taxus* (Hao et al., 2016), *Cornus officinalis* (Sun et al., 2022), *Astragalus mongholicus* (Li et al., 2021), and *Baphicacanthus cusia* (Zeng et al., 2018) differ from *C. migao*. Besides, widespread species in order to adapt to different environments, leading to the creation of stably inheritable variation that results in multiple genotypes of the same species. The chemical components and contents of these genotypes may differ, e.g., the polyphenols and antioxidants contents of Sorghum were higher in two genotypes and simultaneously did not differ significantly between some genotypes (Aruna et al., 2020). Based on the small genetic differentiation among populations of *C. migao* (Li et al., 2018), we speculate that environmental heterogeneity are more likely to be responsible for the differences in active ingredients and dominance of homogeneous genera in *C. migao.* This is substantiated by the reports of others (Li et al., 2016), indicating that differences in environmental conditions, soil properties, and microorganisms can result in substantial differences in the quality of medicinal plant materials.

### 4.2. Effects of soil properties on rhizomicroorganisms

The structures and diversities of microbial community are meaningful indicators for evaluating ecosystem stability (Chapin et al., 2011; Romaniuk et al., 2011; Wigginton and Amador, 2020). Several environmental factors affect soil microbial community structure and diversity, including primarily climatic, phytic, and soil-specific factors (Marschner et al., 2001; Yang et al., 2019; Wang et al., 2021). Of these, soil properties have been found to be the primary driver of microbial community structure (Philippot et al., 2013), particularly soil pH (Scola et al., 2018). Our results are in agreement with these previous studies, indicating that pH is a key driver of rhizobacterial community structure (Jiang et al., 2017). Differences in response patterns of bacteria-specific to pH gradients were observed, for example, *Nitrospira*, *Gaiellales* (unidentified), *Xanthobacteraceae* (unidentified), and H16 were positively correlated with pH, while *Acidothermus*, *Variibacter*, *Bradyrhizobium*, *Acidibacter*, *Acidobacterianace* (unidentified), *Alphaproteobacteria* and *Candidatus-Solibacter* were negatively correlated with pH. This dual response is consistent with other studies (Jones et al., 2009; Dimitriu and Grayston, 2010; Rousk et al., 2010). The acidic soil environment of *C. migao* may provide suitable conditions for acidophiles to colonize and enrich, such as, *Acidothermus*, *Alphaproteobacteria*, *Acidibacter*, and *Acidobacterianace* (unidentified). Additionally, *Nitrospira* and *Xanthobacteraceae* (unidentified) were found to have a close positive correlation with TN and AN, nitrogen levels are also controlling factors for *C. migao* rhizobacteria.

In fact, there is an interaction, soil physicochemical factors not only affect microorganisms, but also microorganisms can react to soil properties. For example, *Acidibacter* as an acidophilic ferric reducing gammaproteobacterium can causing the pH to increase when it catalyzed the reductive dissolution of the ferric iron mineral schwertmannite (Falagán and Johnson, 2014); *Nitrospira* plays pivotal role in nitrification as an aerobic chemolithoautotrophic nitrite-oxidizing bacterium, metabolic versatility enables *Nitrospira* to colonize a broad range of habitats and to sustain shifts in environmental conditions such as changing oxygen concentrations and promoting nitrogen-cycling (Daims et al., 2015; Daims and Wagner, 2018), this is verified by the higher *Nitrospira* abundance and AN contents in LB samples. It’s showed that the enrichment of acidophiles and *Nitrospira* may be primarily responsible for the acidity and nitrogen-rich in *C. migao* rhizosphere soil. The further description is that C-, N- and P-cycles in soil are subject to the allocation of microbial community structures and functions, the growth and activity of endemic rhizomicroorganisms can promote the transformation and storage of effective nutrients in rhizosphere soil, which stimulate the uptake of nutrients by plant roots.

In addition, we found connection between soil pH, soil nutritional properties, and rhizobacterial diversity. On the one hand, pH and soil nutrients are positively correlated, especially with AK, AP and OM, pH can affect the effectiveness of soil nutrients, and it may be a key factor in regulating soil nutrients and promoting plant nutrient uptake. On the other hand, there are also positive correlation among pH, nutrients and rhizobacterial diversity. For example, soils with very low pHs tended to have lower nutritional content and, consequentially, lower rhizobacterial diversity, as was the case for ZF. Contrarily, soils with higher pH tended to have higher nutritional content and rhizobacterial diversity, as was the case for LY. This supported that soils with higher nutrient content tend to have higher microbial diversity (Maestre et al., 2015; Gebhardt et al., 2017), but other samples (WM, CH, LB, etc.) do not follow this universal rule. So other factors affecting rhizomicrobial diversity should be taken into account.

We comprehensively analyzed the geoclimatic information across all sample sites, the results showed that although a geoclimatic analysis of the nine sample sites indicated a significant elevation gradient, no significant correlations were found between elevation and soil properties, rhizobacterial diversity. This is consistent with reports indicating that bacterial community distribution may not follow the same elevational distribution patterns exhibited by plants or animals (Shen et al., 2013), but contrary to other reports indicating that bacterial communities do differ by elevation, despite perhaps indirectly (Fierer et al., 2011). Alternatively, temperature may be an important factor in the differentiation of the rhizobacterial diversity, for example, the low rhizobacterial diversity of ZF may be related to its relatively lower mean annual temperature (17.98°C), and the higher bacterial diversity of WM may be related to its relatively higher mean annual temperature (20.29°C). Soil properties explained 75.54% (RDA1 57.57%, RDA2 17.97%) of the variation in rhizobacterial community structure. This explanation rate was close to apple trees of around Bohai Gulf, with a cumulative explanation rate of 78.88% (RDA1 50.43%, RDA2 28.45%) for RDA (rhizobacteria × soil properties; Jiang et al., 2017), and both major contributing factors included pH and AN; *Betula albosinensis* was 57.43% (RDA1 42.65%, RDA2 14.72%), and its dominating factors showed SOC > C:N > TN > pH (Du et al., 2017). This discrepancy may be related to the different soil factors selected. The explanation rate of 75.54% indicates that soil chemistry is the dominant influence of rhizobacteria in *C. migao*, which may be connected with the existence of long-term, direct × indirect interactions with rhizomicroorganisms in a combination of multiple ways. The related percentage of variation unexplained by factors studied here (24.46%) suggests that there are other environmental or biological factors influencing *C. migao* rhizobacterial community structure. Future studies should examine the effect of other climatic, geographical, soil physiochemical properties (e.g., soil enzymatic activity, aeration, and water permeability), and associated plant communities on *C. migao* rhizobacterial community structure and diversity.

### 4.3. Effects of soil rhizobacteria on medicinal plants

Plant growth-promoting rhizobacteria (PGPR) colonize plant roots and stimulate plant growth, stress tolerance, and defense (Kloepper and Schroth, 1978). The widely reported PGPR in *C. migao* include *Bradyrhizobium* and *Bacillus* (Podile and Kishore, 2006; Lugtenberg and Kamilova, 2009; Haney et al., 2015; Xie et al., 2019), which have disease-preventing and growth-promoting effect on *C. migao*. They promote plant growth by enhancing nutrient acquisition, niche competition, the production of antimicrobial compounds, or inducing systemic resistance to prevent pathogen infection (Zamioudis et al., 2013; Pieterse et al., 2014, 2016; Chowdhury et al., 2015). Recent studies have demonstrated that not only do rhizobacteria and their metabolites affect the growth and stress resistance of plants, but they can also impact plant accumulation and synthesis of medicinal bioactive compounds (Köberl et al., 2013; Huang et al., 2018; Zhang et al., 2020). There are three primary mechanisms by which rhizomicroorganisms affect the accumulation of bioactive secondary metabolites in medicinal plants. First, microorganisms activate secondary metabolite signaling pathways in plants, resulting in the upregulation of secondary metabolite biosynthesis (Trivedi et al., 2020). Second, microorganisms themselves produce bioactive secondary metabolites which stimulate the upregulation of secondary metabolite biosynthesis in plants (Schmidt et al., 2014). Third, microorganisms improve the stress resistance of medicinal plants, which is often mediated by the biosynthesis of secondary metabolites (Taghinasab and Jabaji, 2020). Relevant representative studies, *Piriformospora indica* and *Azotobacter chroococcum* can significantly enhance the artemisinin content of *Arteuaisia annua* (Arora et al., 2016). *Stenotrophomonas maltophilia* and *Pseudomonas fluorescens* can significantly enhance the hypericin and hyperforin content of *Hypericum perforatum* (Manero et al., 2012). *Pseudomonas* can regulate gene expression in *Taxus* cells by releasing the phytotoxin-coronatine, which may be responsible for the resultant increase in taxane content (Onrubia et al., 2013). *Burkholderia* sp. increases the synthesis of indigo in *Baphicacanthus cusia* (Zeng et al., 2018). *Bacillus pumilus* enhances glycyrrhizic acid content by increasing the expression of key enzymes (Xie et al., 2019).

Here, we found that several bacteria, including *Acidothermus*, *Acidibacter*, *Bryobacter*, *Variibacter*, *Candidatus_Solibacter*, and *Acidimicrobiales* (unidentified) were positively correlated with the content of bioactive compounds in *C. migao*. Interestingly, *Acidobacteria* (unidentified) was positively correlated with all compounds except limonene, and *Nitrospira*, *Alphaproteobacteria*, and *Xanthobacteraceae* (unidentified) were negatively correlated with all four bioactive compounds. Another important finding is that the functional classification of *C. migao* rhizobacteria tended to be homogenous across regions, with metabolism-related functions being the most common. Therefore, we speculated that *C. migao* may selectively recruit specific microorganisms in rhizosphere that could metabolize certain substances to effect *C. migao*. Based on the data obtained in this study, the enrichment of bacterial genera *Acidothermus*, *Acidibacter*, *Bryobacter*, *Variibacter*, *Candidatus_Solibacter*, and *Acidimicrobiales* potentially promote the biosynthesis and accumulation of 1,8-cineole, cypressene, limonene, and α-terpineol in *C. migao*, while *Nitrospira* and *Alphaproteobacteria* may be repressive for the bioactive ingredients. However, the mechanism by which these rhizobacteria alter the accumulation of bioactive compounds remains to be identified.

Among the enriched taxa in *C. migao* rhizosphere. *Acidothermus* was widely known as the *Acidothermus celulolyticus* endoglucanase (E1; Larry et al., 2008). It has been recorded to colonize the rhizosphere of Lettuce (Cipriano et al., 2016), and found to be associated with the oxidation of sulfur, nitrogen and iron (Wang et al., 2022). This oxidation alters the uptake of sulfur, nitrogen, and iron by plants, affecting the synthesis of plant metabolites, in this study, *Acidothermus* may be a potentially beneficial bacteria used to promote the accumulation of bioactive compounds (especially limonene) of *C. migao*. *Acidibacter*, *Bryobacter*, *Variibacterr*, and *Candidatus_Solibacter* may have a slight stimulating effect on the accumulation of bioactive ingredients in the fruit of *C. migao. Acidibacter* colonized in many plant species, and associated with soil nutrient and iron cycles, plant blights, and soil pollution controls (Falagán and Johnson, 2014; Liu et al., 2016; Jiao et al., 2018; Huang et al., 2022), it may has a variety of potential functions. *Bryobacter* has been reported as beneficial bacteria in studies such as *leguminous* plants and *Marchantia*, where it plays roles in plant-growth promotion, complex exudate degradation, nitrogen fixation, methylotrophs, and disease-suppressive (Xiao et al., 2017; Luis et al., 2018). *Variibacterr* has been documented in soil contamination and nutrient cycle studies (Jiao et al., 2018; Wu et al., 2020), which may be a microbial community with degradation potential, although it has decreased abundance in tobacco-peanut intercropping soils with *Burkholderia* (Gao et al., 2019), it may also have biocontrol roles as *Burkholderia*. The abundance of *Candidatus_Solibacter* were increased in healthy sesame rhizosphere soils with *Bryobacter* and *Bradyrhizobium*, they were beneficial to improving the soil microecology and alleviating continuous cropping obstacle (Wang et al., 2017). *Alphaproteobacteria* is a diverse class of organisms within the phylum Proteobacteria, its members inhabit diverse environmental niches and have many important biological roles, they participate in a variety of metabolic strategies, including nitrogen fixation, photosynthesis, ammonia oxidation, and methylotrophy (Williams et al., 2007; Andreote et al., 2009; Collier, 2016), in this study, *Alphaproteobacteria* may suppresses the accumulation of *C. migao* bioactive ingredients by participating in a particular metabolisms*. Nitrospira* was widely reported as an aerobic autotrophic nitrite-oxidizing bacteria that plays a key role in plant nitrification and promotes efficient conversion and storage of nitrogen (Daims et al., 2015; Daims and Wagner, 2018); additionally, *Nitrospira* has also been reported to be associated with verticillium and fusarium wilts of cotton and watermelon (Zhu et al., 2019), the complexity of *Nitrospira* functions makes it possible to both as beneficial bacteria to stimulate plant absorption of effective nutrients, as well as a pathogen to induce systemic resistance of plants, *Nitrospira* may inhibit the accumulation of medicinal bioactive ingredients of *C. migao*. Overall, the enriched strains and their metabolites may promote or inhibit the growth and quality of *C. migao* through a variety of pathways. We suggest that the rhizosphere of wild-type-*C. migao* are enriched with a large number of potentially valuable bacteria. It provided a reference for the next step of screening beneficial bacteria to promote the growth and quality of *C. migao*. Meanwhile, the function of specific microbial strains should be validated, and the mechanisms of specific strains on bioactive ingredients in *C. migao* should be elucidated in the future.

The effective magnitude of rhizomicroorganisms on bioactive compounds is likely to be species-, tissue-, and contact-dependent. For example, in herbs with roots as medicinal parts, 57 OTUs are positively (0.9 ≥ R^2^ ≥ 0.51) or negatively (−0.85 ≤ R^2^ ≤ −0.52) correlated with the bioactive ingredient in *Astragalus mongholicus* (Li et al., 2021); 13 rhizobacteria genera are significantly (*P* < 0.05) associated with bioactive ingredient in *Scutellaria baicalensis* (Jiang, 2021). In herbs with the whole plant as medicinal parts, the correlation of bioactive ingredients of different medicinal parts with rhizobacteria is shown as aboveground < underground (R^2^) in *Rumex japonicus* (Ni, 2019). The relationship between three medicinal licorices endophytic bacteria and the bioactive ingredients showed that the cumulative interpretation rate was 22.33% (RDA1 12.55%, RDA2 9.78%; Dang et al., 2020). Combined with our results, soil properties explained 75.54% of the variation in rhizobacterial community structure, whereas rhizobacteria explained 19.69% of medicinal components. (a) We speculated that the above phenomena may be related to the effect of rhizobacteria on the medicinal ingredients in roots or rhizomes of herbs could rely on dual influences of direct and indirect, while fruit trees could basically depend on indirect effects. (b) The bioactive ingredients of fruits and rhizobacteria are not the dominant influencing factors for each other despite they somewhat correlated. The remaining 80.31% of unexplained factors may be related to other unmeasured indicators, such as: temperature, light, moisture, endophytes, other rhizomicroorganisms, and rare genera (relative abundance < 1%). Although the interpretation of interaction between arbor rhizobacteria and fruit medicinal ingredients is limited, it cannot be ignored, which can provide a possibility for understanding the complex ecological characteristics of *C. migao* and developing *C. migao* medicinal resources.

In summary, this study revealed the complex correlation of rhizobacteria with bioactive ingredients and soil properties, which provides us with an opportunity to improve and enhance the quality of *C. migao* from a microbial perspective.

## Data availability statement

The original contributions presented in the study are included in the article/Supplementary material, further inquiries can be directed to the corresponding author.

## Author contributions

BT, XY, DW, XT, and XX: conceptualization. LL, BT, and XY: methodology. BT and JC: validation. LL and BT: formal analysis. BT, DW, and JC: investigation. LL: data curation and writing—original draft preparation. LL and XY: writing—review and editing. JL and SW: visualization. JL: supervision. XY and JL: funding acquisition. All authors contributed to the article and approved the submitted version.

## Funding

The Guizhou Provincial Key Technology R&D Program of China (Qiankehezhicheng[2021]yiban143); National Natural Science Foundation of China (NSFC, 32171511); National Natural Science Foundation of China (NSFC, 31800335).

## Conflict of interest

The authors declare that the research was conducted in the absence of any commercial or financial relationships that could be construed as a potential conflict of interest.

## Publisher’s note

All claims expressed in this article are solely those of the authors and do not necessarily represent those of their affiliated organizations, or those of the publisher, the editors and the reviewers. Any product that may be evaluated in this article, or claim that may be made by its manufacturer, is not guaranteed or endorsed by the publisher.

## Footnotes

^1^ http://www.i-sanger.com/

^2^ http://www.arb-silva.de

^3^ http://www.mothur.org/

## Abbreviations

OM: organic matter
TN: total nitrogen
AN: alkali hydrolyzed nitrogen
TP: total phosphorus
AP: available phosphorus
TK: total potassium, and available potassium (AK)
